# Gastrointestinal manifestations of synthetic cannabinoids: a retrospective cohort study

**DOI:** 10.1186/s12876-021-01847-w

**Published:** 2021-07-06

**Authors:** David Hakimian, Ariel A. Benson, Tawfik Khoury, Muhammad Massarwa, Sarah Israel, Shaden Salameh, Yonatan Gershinsky, Barak Shapira, Mordechai Muszkat

**Affiliations:** 1 Institute of Gastroenterology and Liver Diseases, Hadassah Medical Center and Faculty of Medicine, Hebrew University of Jerusalem, 91240 Ein Kerem, Jerusalem, Israel; 2 Department of Gastroenterology, Galilee Medical Center, Nahariya, Israel; 3 Bar-Ilan Faculty of Medicine, Safed, Israel; 4 Department of Internal Medicine, Hadassah Medical Center Mt Scopus and Faculty of Medicine, Hebrew University of Jerusalem, Jerusalem, Israel; 5 Department of Emergency Medicine, Mt Scopus and Faculty of Medicine, Hebrew University of Jerusalem, Jerusalem, Israel; 6 Braun School of Public Health and Community Medicine, Hebrew University Ein Kerem Medical Campus, Jerusalem, Israel; 7 Division of Enforcement and Inspection, Israel Ministry of Health, Jerusalem, Israel

**Keywords:** Gastrointestinal, Synthetic cannabinoids, Mesenteric event, Abdominal pain

## Abstract

**Background:**

Synthetic cannabinoids (SC) are chemical substances which activate cannabinoid receptors similarly to tetrahydrocannabinol, but with a higher efficacy. These substances are used as illicit recreational drugs, often smoked as herbal mixtures. The continuing availability and rapid evolution of SC is an ongoing health risk. The adverse effects of SC are wide ranging, and span from mild behavioral changes to death. Knowledge regarding gastrointestinal (GI) manifestations of SC use is sparse.

**Methods:**

Single tertiary-care referral medical center retrospective study.

**Results:**

The medical records of patients presented to hospital emergency care due to SC use between January 2014 and February 2018 were retrieved from Hadassah Mount Scopus Hospital’s computerized database. The records were reviewed for clinical outcomes and laboratory tests. Fifty-five (55) patients were identified with a hospital presentation due to SC use. Twenty-one (21) out of 55 patients (38%) reported gastrointestinal complaints. The most common complaints were abdominal pain and vomiting. Of those, 28% had recurrent emergency department presentations due to abdominal pain and 66% presented with leukocytosis. Serum lactate was elevated in 66% of patients with GI manifestations. One patient had an abnormal computerized tomography (CT) abdominal angiography scan, which was compatible with intestinal ischemia.

**Conclusions:**

The clinical spectrum of gastrointestinal manifestations in SC intoxication ranges from mild symptoms, such as abdominal pain and vomiting, to even more severe symptoms suggestive of intestinal ischemia. Clinicians should be aware that abdominal pain and other gastrointestinal complaints can be associated with SC use.

## Background

Since the early 2000s, synthetic cannabinoid compounds (SC), first developed by researchers to study the cannabinoid receptor (CBr), have become popular recreational drugs of abuse mostly due to their psychoactive properties and high potency. SC were first synthesized in the 1960s to explore potential medical uses of compounds designed to target CBr. Over time, SC have been modified and widely distributed for illicit use, with most SC users being male and concurrent users of cannabis [1]. The life prevalence of SC use was demonstrated to be 7.6% in a survey of more than 3,100 high school seniors and college students in the United States [2] and was found to be as high as 17% in an anonymous online survey of over 15,000 participants in the United Kingdom [1].

SC usually appear in the illicit drug market as smokable herbal mixtures containing shrub leaves. These substances can also be consumed via vaporized liquid, inhaled in e-cigarettes, or by ingestion. The herbal mixtures that carry the active SC are sprayed following the dissolution of the synthetic substance in acetone or a similar solvent [3]. New SC are constantly being developed, and in some jurisdictions, new compounds are legal to possess until they are eventually formally banned by law. Due to the ability of laboratories to rapidly change the chemical structure of SC, it is especially difficult to monitor and restrict their use solely by law enforcement agencies [4]. SC products sold on the street and online, are referred to by various names, such as “Spice”, “K2 and “Mr. Nice Guy” [5]. The active chemicals are not characterized using controlled laboratory testing, and many products are mixed with potentially dangerous substances such as other illicit drugs, animal-oriented poisons, or embalming fluids [6]. Thus, the clinical consequences of SC use are myriad, yet not well-defined [7].

Patients admitted to the hospital emergency services due to SC intoxication mostly present with neurological and psychiatric manifestations, such as agitation, psychosis, or anxiety. SC users might also present with seizures [8] (reported to lead to rhabdomyolysis and hyperthermia), acute renal failure [9], or myocardial ischemia, which was observed even in teenagers [10]. The gastrointestinal (GI) effects of SC use have not been thoroughly described. GI symptoms in SC intoxication may be the result of cannabinoid interaction with CBr1 and CBr2 [11]. Of note, CBr1 and CBr2 have been detected in peripheral tissues, including the GI tract [12].While reports on the epidemiology and clinical spectrum of GI manifestations of SC use are limited, cases have demonstrated that SC can exert their effects on the GI tract, causing varying symptoms such as vomiting and abdominal pain [7]. SC use can also lead to a hyperemesis syndrome similar to that observed in cannabis use [13]. In this study, we examine the association between SC use and the GI tract manifestations, using a cohort of patients which presented to the hospital after SC use. Furthermore, we aim to understand the clinical impact, pathophysiology, and prognosis related to SC use with GI manifestations.

## Methods

Electronic medical records of patients admitted to a single tertiary-care referral medical center between January 2014 and February 2018 were retrospectively reviewed. The electronic medical record database was searched for the keywords "synthetic cannabinoids" and "Nice Guy" (The most commonly used SC product in Israel). The medical records of patients admitted who reported SC use were then reviewed and data was collected, including demographics, clinical and laboratory results, reason for hospital admission, length of hospital stay, placement in the hospital, diagnostic tests for SC levels (i.e., Gas Chromatography-Mass Spectrometry (GC–MS) urine tests for synthetic cannabinoids and other unknown materials), imaging tests (including computed tomography (CT), and patient outcomes. Records were also reviewed for GI manifestations associated with SC use.

Patients were excluded if they were younger than 18 years old, had a confirmed diagnosis of an active psychiatric disorder, or had a known chronic GI disorder. GI manifestations were defined as symptoms of abdominal pain, vomiting, or diarrhea that were reported in the emergency department (ED), as documented in the medical records. The study was approved by the hospital ethics committee and was exempt from patient consent given the retrospective nature of the study and that data was stored anonymously.

**Fig. 1:**
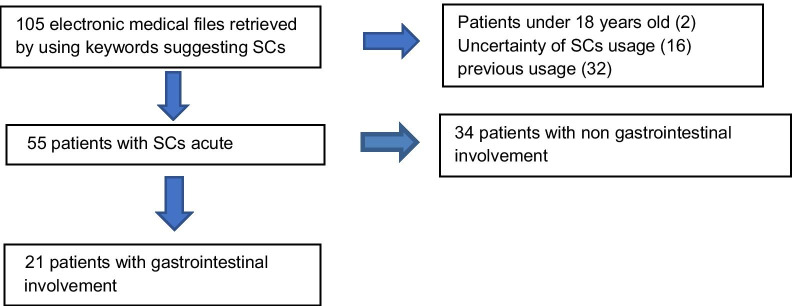
Flow diagram of patients selected for study (N = 105)

One hundred and five (105) patients with reported SC use were identified. Two of the patients were excluded as they were under the age of eighteen. Additional 16 patients were excluded due to uncertainty regarding their SC use in the medical chart. Another 32 patients were excluded because exposure to SC was reported as a part of prior medical history, rather than being part of the current hospitalization. The remaining 55 patients were included in the study (Fig. 1).

### Statistical analysis

We calculated descriptive statistics for demographic, and treatment related characteristics in the study population. These included: age, sex, hospital attendances, hospitalizations, and length-of-stay. We also calculated descriptive statistics for a range of relevant clinical biomarkers. Continuous variables are presented as mean, and standard deviation. We used independent-samples T-test to explore differences between patients with, and without GI symptoms on all continuous variables. The T-test inferential statistic was used despite deviations from normalcy because of its robustness and a sample size that was deemed sufficient. A *p* value of < 0.05 was considered statistically significant. Statistical analyses were performed using Microsoft Excel 2016 (Redmond, WA) and R version 4.0.3 (R Core Team).

## Results

Twenty-one (21) out of the 55 patients included in the analysis (38%) were admitted with GI symptoms. Additional common symptoms leading to admission were chest pain, nausea, agitation, and restlessness. Abdominal pain was the primary reason for presentation in 18 of the 21 patients (86%) with GI manifestations and 12 of the patients (57%) reported vomiting. Repeated ED admissions due to GI symptoms were noted in 28% (n = 6) of patients, and, out of these, two patients had seven prior admissions to the ED due to various complaints related to SC use. Of those with GI manifestations secondary to SC use on ED presentation, 24% (n = 5) were hospitalized due to these GI symptoms, while the rest were discharged from the ED for outpatient follow-up. Demographic and treatment related data on patients is presented in Table 1.

**Table 1:** Demographic and treatment-related characteristics of patients in those with and without GI symptoms

N (%)	GI symptoms (n = 21)	No GI symptoms (n = 34)
Male	20 (95)	29 (85)
Hospitalized patients	5 (24)	9 (27)
**Median (IQR)**		
Age	28 (14)	35 (21)
Hospital attendances	1 (2)	1 (3)
Duration of hospitalization^*^	3 (4)	8 (7)

GI: gastro-intestinal

^*^In hospitalized patients

Two of the hospitalized patients in the GI group were admitted to the intensive care unit, and both had multiple prior presentations to the ED secondary to SC use. One of these patients died, likely due to small intestinal perforation leading to severe shock—see below. The second patient presented with severe hypokalemia and a potassium of 1.7 mEq/L (normal range 3.5–5.1 mEq/L).

Ten patients of those with GI symptoms had blood gas testing performed in the Emergency Department, two of whom had a metabolic acidosis, one had metabolic alkalosis, and one had a respiratory alkalosis. The remaining six who underwent blood gas testing were found to have blood gas levels within the normal range.

All 21 patients with GI manifestations underwent complete blood count laboratory testing, 16 of whom (76%) had leukocytosis (normal range: 4–10E9/L), with six patients (28%) having leukocyte levels greater than 20 E9/L. Serum lactate (normal range: 0.5–2.4 mmol/L) was measured in eight patients (38%) in those with GI manifestations and was measured in six (17%) patients in those without GI manifestations. Lactate was elevated in six patients (66%) in those with GI manifestations and in two patients (33%) in those without GI manifestations. In two patients with GI manifestations, serum lactate was severely elevated (greater than eightfold higher than the upper limit of normal range) (Table 2).

**Table 2:** Clinical and Laboratory results of patients with GI and without GI presentation of SC intoxication

Measure (mean ± SD)	With GI symptoms	Without GI symptoms	*p* value
Temperature	36.5 ± 0.6	36.6 ± 0.5	0.50
Heart rate	72.9 ± 20.8	79.3 ± 17.3	0.26
Systolic BP	131 ± 18.6	134.2 ± 18.5	0.84
WBC	17.2 ± 8.3	14.0 ± 5.6	0.09
HGB	16.3 ± 1.3	15.6 ± 1.7	0.19
ALT	22.2 ± 14.5	41.0 ± 68.9	0.30
ALK PHOS	81.9 ± 24.0	91.8 ± 36.8	0.37
pH	7.4 ± 0.1	7.3 ± 0.2	0.19
Lactate	4.3 ± 4.9	4.5 ± 4.4	0.92
Creatinine mmol/Liter	104.8 ± 60.3	85.1 ± 26.0	0.24
Potassium mEq/Liter	3.7 ± 0.8	3.8 ± 0.5	0.50

One third of the patients with GI symptoms (n = 7) underwent an abdominal computed tomography (CT), of which six were normal and only one was abnormal, showing thromboses in the splanchnic arteries, mesenteric ischemia, and bowel perforation. Half of the patients with GI symptoms (n = 11) underwent standard urine toxic screen examination. In four (36%) of these patients the test was positive for THC, in four (36%) patients the test was positive for amphetamines, in two (16%) patients the test was positive for benzodiazepines, and in two (16%) patients the test was positive for methadone traces. Four patients (36%) had a negative urine toxicology screen. None of our patients underwent GC–MS testing in the hospital at the time of their presentation.

## Discussion

This is, to our knowledge, the first study specifically dealing with the GI symptoms of SC use in an acute setting. Among symptomatic SC users presenting to the ED, 38% had GI manifestations and abdominal pain was reported by of those with GI symptoms. Still, the clinical presentation, laboratory results, and imaging findings ranged in severity among these patients. Most patients had only mild abdominal pain, while others had severe elevations of serum lactate with one patient having suffered an intestinal perforation. Nevertheless, the vast majority of patients (20/21) had a complete resolution of abdominal pain and normalization of serum lactate levels within a few hours of presentation.

When comparing the differences between the patients with GI symptoms and those without, there were no statistically significant differences in their prognoses, clinical severity, or laboratory values. Lack of significant difference may have been due to their low numbers. However, the patients with GI manifestations had higher lactate levels likely caused by the intestinal involvement and more patients with severe leukocytosis. The mechanism causing the varied effects of SC on the GI tract is unclear, but may be related to a previously reported effect of THC and cannabidiol on CBr1 and CBr2, which influence GI function, motility, and sensation [14, 16]. Our study suggests that SC GI symptoms are unpredictable and can vary from minimal, or no symptoms to intestinal perforation and death [15]. This varied GI response to SC may be due to the varying potency of SC, especially with the development of new ultra-potent SC that may impact the GI tract more than regular cannabis [17]. Others have suggested that the variability of SC on the GI tract may be due to adulterants added to the compounds (such as caffeine, nicotine, and tramadol), which can contribute to clinical effects and toxicity [18, 19].

An additional proposed mechanism of SC induced GI symptoms may involve vascular spasm. SC have been shown to rapidly alter neurotransmitter release from nerve terminals, thereby potently activating vascular smooth muscle cells, potentially resulting in vascular spasm. Rose et al. reported two cases of subarachnoid hemorrhage following SC consumption and used digital subtraction angiography to confirm transient vasospasm [20]. Moreover, Mir et al. reported two patients with ST-elevation MI following SC use with subsequent normal coronary angiography [21]. These reports imply that SC causes vasospasm in other organs without solid evidence regarding the GI tract. The low proportion of abnormal imaging in patients with severe abdominal pain, as in our study, supports the vasospasm hypothesis as vasospasm is reversible, and no vascular pathological findings were demonstrated. This assumption is less established and controversial.

Currently, with the increasing prevalence of cannabis use by the public, there is extensive awareness among clinicians of the GI symptoms related to cannabis use, including abdominal pain and vomiting [22]. However, SC related GI symptoms have been less frequently discussed. Nausea and vomiting and abdominal pain are frequent features of SC presentations [7, 23]. Treatment is mostly symptomatic, and involves intravenous hydration, and the use of anti-emetics [13, 24]. In other cases, treatment requires the use of sedatives and antipsychotics [13] The lack of identifiable SC in the toxicology screening in routine use make the diagnosis of SC related GI symptoms difficult.

One limitation of our study is a lack of diagnostic serum and urine analytical studies to diagnose objective SC use, a challenge noted in many SC cohorts. As in many hospitals, the availability of gas chromatography and mass spectrometry testing in the acute setting is lacking, and similar to other studies in this field. We currently rely on patient and/or family reported history. A second limitation of our study is that patients presenting with SC use often have a concomitant drug intoxication, such as amphetamines and cannabis, which can also impact GI symptoms in a similar manner although not in the same rates of GI involvement compared to SC. A third limitation is the lack of autopsy and post mortem imaging aside from one post mortem CT. Notwithstanding these limitations, we present the first cohort of patients presenting with various GI symptoms and SC use in the acute setting. This can help to raise awareness and potentially guide future studies to evaluate the mechanisms of SC GI involvement, including the hypothesis that transient arterial vasospasm causes the acute GI symptoms. GI consequences occurring with SC use are often short-lived and resolve quickly, although in certain instances these can be life-threatening (e.g. intestinal perforation; as demonstrated by one case in this cohort).

Our findings have a number of implications on the treatment of SC related intoxication. First, they emphasize that awareness of the breadth of clinical presentations and GI signs/symptoms common to SC intoxication is important for healthcare providers treating these patients. This is particularly important when practicing in areas where there is a high prevalence of SC use. As SC use is not associated with a well-defined toxidrome, some clinicians are less confident in managing their acute toxicity [25]. Second, clinicians should be aware of the range, and dynamic nature of SC related GI manifestations. Initial bouts of abdominal pain, nausea and emesis could well develop into fatal mesenteric ischemia, which necessitates surgical intervention. Although SC toxicity appears to be involved in the suppression of symptoms of intestinal ischemia or may even exacerbate existing ischemia, it is not possible with current evidence to ascertain that it is the cause of ischemia. However, the fact that our study did not demonstrate significant differences in GI involved biomarkers and non-GI involved cases of SC consumption suggests that clinicians should be overtly attentive to the challenge of identifying at-risk patients. Hence, our study may aid in helping recognize further complications of SC use to facilitate the diagnosis of intoxications, and their treatment.

Better analytical testing and drug confirmation techniques are needed along with prevention of SC use via counseling in at-risk populations is vital to reduce public health implications and morbidity from SC use.

## Conclusions

Physicians and clinicians in the acute setting should consider SC use as an etiology for unexplained vomiting and severe abdominal pain in young patients, especially when illicit drug use is suspected.

## Data Availability

The data that support the findings of this study are available on request from the corresponding author, {D.H] who received permission from the Hadassah medical center ethical committee. The data are not publicly available due privacy and ethical concerns by the Hadassah medical center Helsinki committee.

## Abbreviations

SC: Synthetic cannabinoid
CBr: Cannabinoid receptor
THC: Tetrahydrocannabinol
GI: Gastrointestinal
CT: Computed tomography
ED: Emergency department
